# Test-retest reliability of the isometric contraction test (IC test) of the masticatory muscles in subjects with and without temporomandibular muscle disorders

**DOI:** 10.1590/1678-7757-2023-0045

**Published:** 2023-10-27

**Authors:** Marcos IGLESIAS-PEÓN, Juan MESA-JIMÉNEZ, César FERNÁNDEZ-DE-LAS-PEÑAS, Nuria GARCÍA IGLESIAS, Carmen María IGLESIAS PEÓN, Daiana Priscila RODRIGUES-DE-SOUZA, Francisco ALBURQUERQUE-SENDÍN

**Affiliations:** 1 Osteopatía y Fisioterapia Guadalajara Guadalajara España Osteopatía y Fisioterapia Guadalajara, Guadalajara, España.; 2 Universidad de Córdoba Programa de Doctorado en Biomedicina Córdoba España Universidad de Córdoba, Programa de Doctorado en Biomedicina, Córdoba, España.; 3 Universidad CEU San Pablo Departamento de Fisioterapia Madrid España Universidad CEU San Pablo, Departamento de Fisioterapia, Madrid, España.; 4 Universidad Rey Juan Carlos Departamento de Fisioterapia, Terapia Ocupacional, Rehabilitación y Medicina Física Alcorcón Madrid España Universidad Rey Juan Carlos, Departamento de Fisioterapia, Terapia Ocupacional, Rehabilitación y Medicina Física, Alcorcón, Madrid, España.; 5 Universidad de Córdoba Facultad de Medicina y Enfermería Departamento de Enfermería, Farmacología y Fisioterapia Córdoba España Universidad de Córdoba, Facultad de Medicina y Enfermería, Departamento de Enfermería, Farmacología y Fisioterapia, Córdoba, España.; 6 Instituto Maimónides de Investigación Biomédica de Córdoba Córdoba España Instituto Maimónides de Investigación Biomédica de Córdoba (IMIBIC), Córdoba, España.

**Keywords:** Temporomandibular disorder, Orofacial pain, Muscle disorder, Myofascial pain, Muscle test

## Abstract

Recently, the DC/TMD has become an essential tool for the diagnosis of temporomandibular disorders (TMD). However, as they fail to include functional activities, new assessment proposals have emerged, such as the isometric contraction test (IC test) of the masticatory muscles, which uses muscle contractions to identify muscular TMD.

**Objective:**

This study aimed to determine the test-retest reliability of the IC test.

**Methods:**

A total of 64 participants (40 women and 24 men) completed the IC test administered by two different physical therapists on two non-consecutive days. Cohen’s kappa (k), PABAK, and percent agreement (PA) between days were estimated.

**Results:**

The IC test showed good to excellent test-retest reliability values (k>0.77; PABAK>0.90), both globally and individually for the muscles evaluated, and PA>90%, therefore above the thresholds for clinical applicability. However, the global assessment of myofascial pain and the evaluation of the medial pterygoid muscle showed slightly lower reliability values.

**Conclusion:**

The IC test is reliable for the assessment of subjects with muscular TMD, both in terms of the global assessment and the evaluation of each muscle, which supports its clinical applicability. Care should be taken when assessing myofascial pain globally and when evaluating the medial pterygoid in all types of pain.

## Introduction

Temporomandibular disorders (TMD) are considered one of the most common pains of non-dental origin.^1^ Population studies have shown that TMD affects 10% to 15% of adults,^2^ with a peak incidence at ages 20 to 40 years, and a similar prevalence in both sexes.^3^ Moreover, TMD accounts for 17,800,000 lost working days per year for every 100,000,000 workers in the United States.^4^

After years of using different classifications for TMD, new related factors, such as epigenetics and neuroscience, and new diagnostic tools have emerged,^5^ such as the diagnostic criteria for TMD (DC/TMD).^4^ DC/TMD Axis I classifies TMD according to the presence of pain (e.g., muscular, of joint origin, and headache attributed to TMD), as intra-articular or degenerative joint disorders or subluxation, and also incorporates new tools in Axis II, which considers various psychological factors that contribute to pain experience, psychosocial aspects, disability, and impaired function.^4^ However, the DC/TMD is questioned, especially due to its dependence on the manual pressure exerted by the examiner during the clinical assessment and the fact that they are not based on functional activities of the masticatory structures.^6^ In fact, the reliability for determining pain in patients with myogenic pain has been considered low,^7^ and recent studies have also questioned manual palpation for the diagnosis of arthralgia in the DC/TMD.^8^ Thus, previous research on the evaluation of TMD based on the combination of dynamic and static evaluations, both at joint and muscle level, has shown high reliability values and a better ability to identify different types of TMD, since pain on palpation is common, even in healthy people.^9,10^

It is therefore necessary to explore further possibilities for assessing DC/TMD Axis I. New diagnostic tests have emerged, such as the isometric contraction test (IC test) of the masticatory muscles,^11^ which has recently been validated in subjects with muscle-associated DC/TMD, both globally (myalgia) and by diagnostic subgroups (local myalgia, myofascial pain, and referred myofascial pain). This test does not require the examiner to palpate the patient, nor does it rely on an experienced examiner to diagnose DC/TMD Axis I of muscular origin. It depends on the muscle pain perceived by the patient when performing the contraction. However, there is no information on the reliability of the IC test or the quality of the results according to the muscle causing the pain symptoms.

It has been reported that the reliability of functional tests for TMD increases significantly when the examiners have been previously trained.^12^ Moreover, it is important to base these diagnoses on previous pain experiences.^7,13^ Consequently, functional tests for temporomandibular pain may be a good option to relate this diagnosis to the pain complaints reported by the patient in the clinical history.^9^

Thus, this study aimed to determine the test-retest reliability of the IC test in subjects with and without DC/TMD Axis I of muscular origin, both globally and according to the type of pain and the muscle causing the symptoms.

## Methodology

### Design

This research consisted of a test-retest clinical study to characterize a diagnostic test, with non-probabilistic recruitment of consecutive cases. All participants signed an informed consent form. Moreover, the study was approved by the Research Ethics Committee of the University of Córdoba (protocol code 5372-2022).

### Subjects

Patients with DC/TMD Axis I of muscular origin and healthy controls of both sexes were recruited from the Health Campus of the University of Córdoba and a private physical therapy clinic in Guadalajara, Spain. Three different recruitment strategies were used: 1) patients who requested treatment and expressed temporomandibular pain in their medical history; 2) patients with a known history of TMD pain who were contacted; 3) patients who responded to advertisements on social networks.

Inclusion criteria were subjects aged 20 to 65 years, of both sexes, with pain in the masticatory muscles for more than 30 days, or the absence of pain in subjects included in the control group. Exclusion criteria were edentulous individuals and patients with temporomandibular joint (TMJ) blockage, acute dental pathology that prevented them from performing the IC test, difficulty understanding basic commands, or who had undergone TMJ surgery in the last 30 days. Moreover, the subjects in the control group were matched to the cases according to sex, age (±5 years old), and BMI (±3 kg/m^2^).

The sample size was estimated using WinPEPI^®^ software, based on an alpha of 5%, with a frequency of positive results of 50% and an expected Cohen’s kappa of 0.8 with a 95% confidence interval (CI) amplitude of 0.3, which generates a lower limit of the interval of 0.6^14^, requiring 64 subjects in total.

### Procedures

After signing the informed consent form, participants completed the validated Spanish version of the DC/TMD Symptom Questionnaire (SQ)^15^ and were classified into the control group and the case group. Then, a clinical examination was performed in search of Axis I diagnoses of muscular origin, according to the DC/TMD protocol.

**Figure 1:**
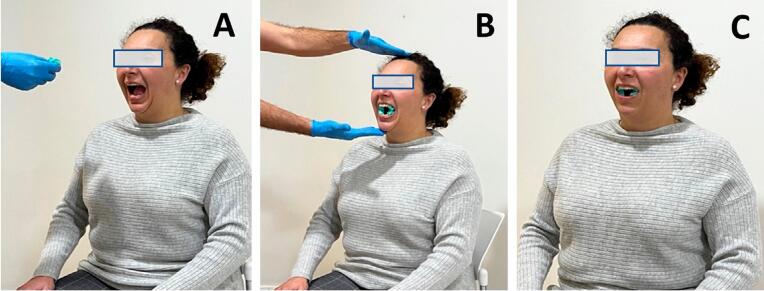
Isometric contraction test of the masticatory muscles. (A) The subject opens the mouth; (B) The examiner inserts the teethers in both sides of the subject’s mouth; (C) The subject clenches the teethers for 40 seconds using maximum pressure

The IC test was administered to all subjects. The examiner, a physical therapist, asked patients to sit on a chair with their feet flat on the floor and their back and head straight. Two teethers (Morde Block size S, Bader Lab, reference 11/022) were placed between their upper and lower premolar and molar teeth, one on each side. Patients were asked to perform a maximal contraction of the occlusal masticatory muscles and to clench the teethers as hard as possible for 40 seconds. They were informed that if they experienced unbearable pain at any time during the test, they should stop the test immediately. After 40 seconds, patients were asked to point whether pain had appeared, the location of the pain, and then whether the pain reminded them of pain suffered in the last 30 days^11^ (Figure 1).

The IC test was considered positive for myalgia when, firstly, pain appeared during the test and, secondly, patients recognized this pain as similar to a pain experienced in the last 30 days. The test was considered negative when there was no pain during the test or when the pain was unknown to the patient. If the IC test was considered positive, the same descriptions established by the DC/TMD were used to assess the type of myalgia (local myalgia, myofascial pain, or referred myofascial pain)^11^ for each muscle evaluated (masseter, temporalis, or medial pterygoid). Local myalgia was considered a pain that arises in the anatomical area of the muscle and therefore does not extend beyond the anatomical border of the muscle evaluated.^16^ Myofascial pain is a spread (but not referred) pain that does not extend beyond the anatomical edge of the muscle, but extends beyond the area of stimulation, considering the area of stimulation as that corresponding to the muscle motor points. Finally, referred myofascial pain was characterized by the existence of referred pain, which extends beyond the anatomical border of the muscle.^4,15,17^ To identify which of the evaluated muscles caused the referred pain, previously published maps were used.^18^

After two to seven days, the IC test was administered again by another examiner, also a physical therapist, to avoid possible bias due to memory of the patient’s clinical status. Both physical therapists, with more than five years of experience in TMD, were trained for 10 hours in the application and interpretation of the DC/TMD and the IC test. Both examiners studied the response to each type of myalgia independently for each muscle (local myalgia, myofascial pain, and referred myofascial pain) and for each of the muscles evaluated (masseter, temporalis, and medial pterygoid) separately.

### Statistical analysis

Results were expressed as mean, standard deviation, and 95%CI for quantitative data and frequencies and percentages for qualitative data. The Kolmogorov-Smirnov test was used to assess the normality of the data. To analyze the differences in the sociodemographic data of both groups, unpaired two-tailed Student’s t-test was used for body mass index (BMI) and the Mann-Whitney U test for age.

Since the kappa value is sensitive to imbalances in prevalence and bias, Cohen’s kappa (k), prevalence-adjusted bias-adjusted kappa (PABAK), and percentage agreement (PA) were estimated to analyze the inter-day test-retest reliability of the IC test, globally (myalgia) for each type of pain (local myalgia, myofascial pain, and referred myofascial pain) and for each muscle evaluated (masseter, temporalis, and pterygoid). The IC test was considered positive when at least one of the muscles, regardless of side, showed pain. Similarly, if the patient expressed pain in more than one muscle, only the muscle that the patient indicated as the most painful was considered in the analysis.

PA is the ratio of the sum of concordant assessments divided by the number of subjects (PA>70% for clinical practice),^19^ whereas PABAK considers unbalanced agreement category scores (prevalence) and differences in proportions of positive and negative results (bias)^20,21^, which negatively affect overall kappa statistics.^22^ For the kappa and PABAK indices, 0 represented no reliability; 0.01 to 0.20 was weak; 0.21 to 0.40 fair; 0.41 to 0.60 moderate; 0.61 to 0.80 good; and 0.81 to 1.00 excellent.^22^

All statistical tests used a 95%CI and a significance value of p<0.05. Data were analyzed using IBM-SPSS (version 25.0; SPSS, Chicago, Illinois), WinPEPI^®^ (J.H. Abramson, August 23, 2016), and PAIRSetc version 3.59 (for 2x2 categories tables).

## Results

**Figure 2:**
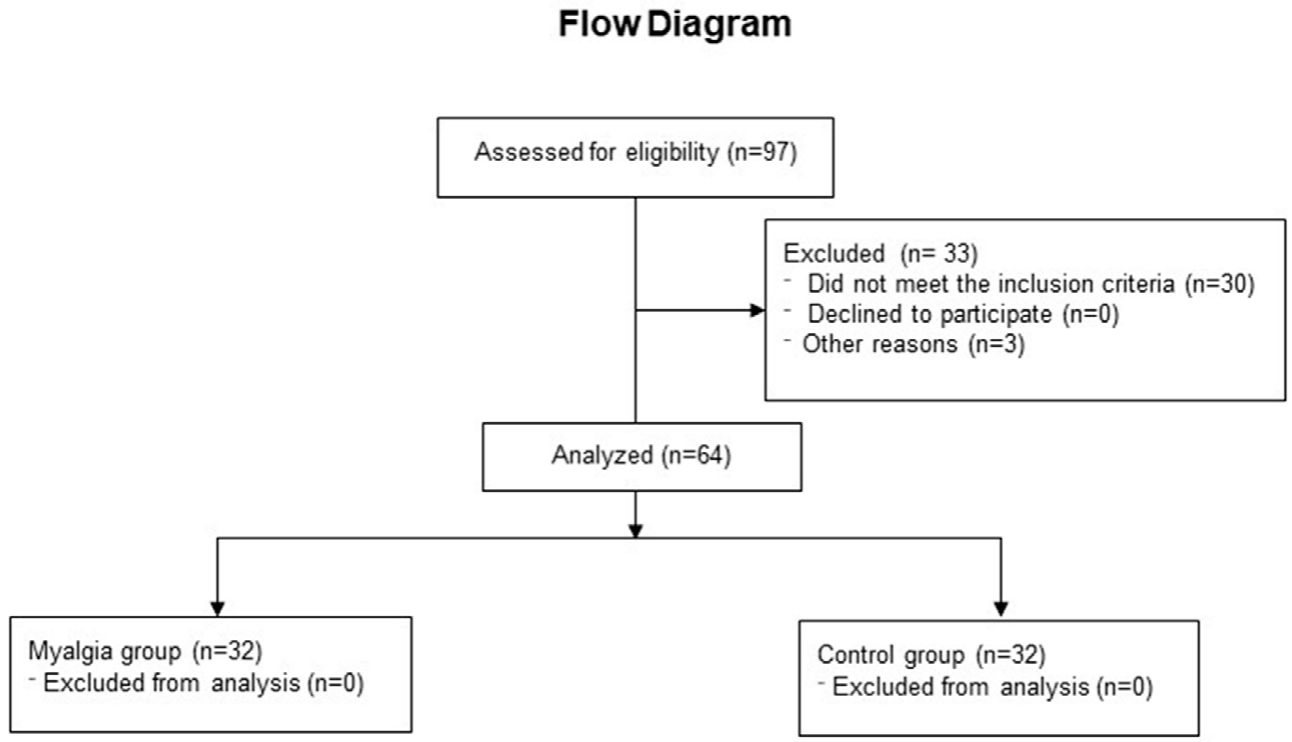
Flowchart of the study

In total, 97 individuals were assessed and 33 were excluded for different reasons. Of the remaining 64, which included 40 women and 24 men, 32 were considered cases of TMD, according to the DC/TMD, and the other 32, who did not meet the inclusion criteria, were control individuals (Figure 2). Age and BMI, which had normal mean values (20–25 kg/m^2^), showed no statistically significant differences between groups (p>0.05). The average duration of the patients’ pain was chronic, exceeding an average of seven years, although there was some asymmetry towards higher values (Table 1). No individual reported unbearable pain or had to abandon the IC test at any time during the evaluation.

**Table 1:** Sociodemographic and clinical data of the sample

	Myalgia group (n=32)	Control group (n=32)	p-value
Age (years)	46 (13)	46 (12)	0.861
Sex (Female/Male)	20/12	20/12	
BMI (kg/m^2^)	24.98±4.45	23.99±3.00	0.295
Time in pain (years)	7.08±8.3		

Values expressed as frequencies, mean±SD, or median (interquartile range).

*Significant difference (p<0.05) between groups. Abbreviations: BMI: body mass index.

The reliability values of the IC test, when individual muscles were not considered, were excellent for the kappa and PABAK indices (≥0.90), for both myalgia and local myalgia. However, myofascial pain showed slightly lower kappa values, ranging from 0.77 to 0.87. PA exceeded 90% in all cases and were therefore above the threshold for clinical applicability (Table 2).

**Table 2:** Interexaminer reliability of the IC test according to the type of pain

	MYALGIA						LOCAL MYALGIA						MIOFASCIAL PAIN						REFERRED MIOFASCIAL PAIN					
	Contraction Day 2						Contraction Day 2						Contraction Day 2						Contraction Day 2					
	Negative	Positive	Total	Kappa	PABAK	PA	Negative	Positive	Total	Kappa	PABAK	PA	Negative	Positive	Total	Kappa	PABAK	PA	Negative	Positive	Total	Kappa	PABAK	PA
Contraction Day 1	32	2	34	0.94 (0.85- 1.00)	0.94 (0.85- 1.00)	96.9 (88- 99)	50	1	51	0.90 (0.77- 1.00)	0.94 (0.85- 1.00)	96.9 (88- 99)	48	3	51	0.77 (0.57- 0.96)	0.84 (0.71-0.98)	92.2 (81- 97)	57	1	58	0.82 (0.57- 1.00)	0.94 (0.85- 1.00)	96.9 (88- 99)
	0	30	30				1	12	13				2	11	13				1	5	6			
Tota	32	32	64				51	13	64				50	14	64				58	6	64			

Values expressed in a 2x2 table as frequencies, kappa, and PABAK. Index value (95%CI). Abbreviations: PABAK: prevalence-adjusted bias-adjusted kappa; PA: agreement percentage.

For each muscle evaluated, all PABAK values were excellent (>0.90), with lower limits of 95%CI ≥0.80, and all PA were >95%, with lower limits of 95%CI ≥0.85, both for myalgia and by type of muscle pain. The medial pterygoid muscles showed the lowest kappa values (0.66<k<0.85) compared with the same type of pain in other muscles, with all lower limits of 95%CI at fair reliability values (lower limit of 95%CI <0.6). For all other muscles and all types of pain, reliability was excellent (k>0.81), except for local myalgia of the temporalis muscle, which was good (k=0.7), as shown in Table 3.

**Table 3:** Interexaminer reliability of the IC test according to the type of pain and the affected muscle

		Myalgia						Local myalgia						Miofascial pain						Referred miofascial pain					
		Contraction Day 2						Contraction Day 2						Contraction Day 2						Contraction Day 2					
		Negative	Positive	Total	Kappa	PABAK	PA	Negative	Positive	Total	Kappa	PABAK	PA	Negative	Positive	Total	Kappa	PABAK	PA	Negative	Positive	Total	Kappa	PABAK	PA
Masseter	Contraction Day 1	37	2	39	0.94 (0.85-1.00)	0.94 (0.85-1.00)	96.9 (88-99)	51	1	52	0.95 (0.85-1.00)	0.97 (0.91-1.00)	98.4 (90-99)	54	2	56	0.87 (0.70-1.00)	0.94 (0.85-1.00)	96.9 (81-97)	58	1	59	0.90 (0.71-1.00)	0.97 (0.91-1.00)	98.4 (88- 99)
		0	25	25				0	12	12				0	8	8				0	5	5			
	Total	37	27	64				51	13	64				54	10	64				58	6	64			
Temporalis	Contraction Day 1	48	2	50	0.87 (0.72-1.00)	0.91 (0.80-1.00)	95.3 (86-98)	61	0	61	0.79 (0.40-1.00)	0.97 (0.91–1.00)	98.4 (90-99)	53	2	55	0.81 (0.61-1.00)	0.91 (0.87-1.00)	95.3 (86-98)	60	1	61	0.85 (0.56-1.00)	0.97 (0.91-1.00)	98.4 (90-99)
		1	13	14				1	2	3				1	8	9				0	3	3			
	Total	49	15	64				62	2	64				54	10	64				60	4	64			
Medial pterygoid	Contraction Day 1	57	1	58	0.70 (0.38-1.00)	0.91 (0.80-1.00)	95.3 (86-98)	62	0	62	0.66 (0.04-1.00)	0.97 (0.91–1.00)	98.4 (90-99)	62	1	63	0.66 (0.04-1.00)	0.97 (0.91-1.00)	98.4 (90-99)	60	0	60	0.85 (0.56-1.00)	0.97 (0.91-1.00)	98.4 (90-99)
		2	4	6				1	1	2				0	1	1				1	3	4			
	Total	58	5	64				63	1	64				62	2	64				61	3	64			

Values expressed in a 2x2 table as frequencies, kappa and PABAK. Index value (95%CI). Abbreviations: PABAK: prevalence-adjusted bias-adjusted kappa; PA: agreement percentage.

## Discussion

This study found that the IC test, used to assess muscular TMD, has good to excellent test-retest reliability. However, lower reliability values were obtained when evaluating myofascial pain without dividing it by each of the muscles evaluated. Similarly, the reliability of the test was lower when the internal pterygoid muscle was evaluated. No patient experienced pain that required them to stop the IC test and, as there were no dropouts, all patients completed the study. Although previous studies have suggested that biting an external element may not be an appropriate way of assessing TMD due to the possibility of causing dental pain,^12^ the test does not depend on an examiner exerting muscular resistance, since the subject who exerts maximum pressure on the teether. Therefore, we recommend the IC test in the clinical setting for the evaluation of DC/TMD Axis I due to its safety, speed, and low cost.

Data show that the test-retest reliability of the IC test has high kappa, PABAK, and PA values, both globally and for each type of pain, when not considering each of the muscles evaluated, which supports its stability over time and regardless of the examiner. However, the lowest values were found for myofascial pain and referred myofascial pain, with good to excellent reliability. In fact, referred muscle pain is considered more complex, both in its origin and its characteristics, compared with local myalgia.^26^ The origin of myofascial pain syndromes and their most frequent clinical presentation, myofascial trigger points, is unknown, although current theories stablish a relationship between peripheral and central sensitization.^17,27^ These associations could involve some parts of the microvascular system and even cellular neurotransmitters.^28,29^ These mechanisms are poorly understood,^26,30^ but some theories support the role of fascial structures in myofascial and referred myofascial pain.^31^ Moreover, this type of pain is linked to different individual perpetuating or stimulating factors, such as stress,^32^ sleep disturbances, or serum inflammation.^33^ Perhaps this component of the individualization of pain, related to many personal aspects, could be the cause of the greater heterogeneity in the triggering of myofascial pain when muscle contraction is requested. In short, it seems that local myalgia is more easily detectable on muscle contraction than myofascial pain or referred myofascial pain.

Reliability values were higher when positive or negative tests were considered at a general level rather than when discriminated by muscle, probably due to the lower number of positives that can be observed when analyzing each muscle unit, which is associated with lower reliability values.^12^ However, among the muscles analyzed, the medial pterygoid had lower reliability values than the masseter and temporalis muscles, regardless of the type of pain reported by patients, with regular values within the 95%CI. This may be because the number of patients who experienced pain in these muscles during the IC test was the lowest of the muscle units and therefore the reliability values obtained should be considered with caution. Moreover, the activation of the medial pterygoid depends on the mandibular opening and its axis of rotation,^34^ which may have conditioned its activation due to the standardization of the IC test and the application of the teether. Thus, the medial pterygoid shows heterogeneous activity in mandibular occlusions,^35^ with different muscle fibers being activated depending on the direction of the contraction requested.^36^ Future studies should assess the reliability of the IC test in identifying changes in the medial pterygoid in different mandibular opening and rotation positions.

In general, the IC test is valid for the diagnosis of myalgia in TMD pain,^11^ with high reliability values, above the thresholds for clinical applicability.^19,22^ The high reliability of the IC test may be due to the low intervention of examiners when applying the test, which eliminates subjectivity in their interpretation of the test.

### Strengths and limitations

The strengths of this study show that the IC test is easy to teach, learn, and perform, and does not require the intervention of an examiner, avoiding contact with the patient, thus differentiating it from other tests in which the results can be compromised due to the influence of examiners and their training.^12^ Regarding the limitations of this study, its results can only be extrapolated to populations similar to the study sample, who did not have temporomandibular joint dysfunction at the time of the study and had a history of temporomandibular pain of muscular origin for at least one month. The results cannot be applied to patients with dental problems or acute pain of dental origin that prevent them from performing a maximum contraction of the masticatory muscles. Moreover, the test result could have been influenced by other pathological conditions, such as stress, rest, inflammation, among others,^32,33^ or the patient’s sensitization to pain at the time of the test,^37^ aspects that were not controlled in this study. Finally, as the IC test is a bilateral assessment of muscles that are anatomically and biomechanically symmetrical during the requested contraction, an individual and separate assessment of each muscle on each side was not performed. Therefore, further studies are needed, with different samples and considering the individual aspects of the subjects that can modify the expression of muscle pain.

## Conclusion

The IC test of the masticatory muscles is reliable in healthy subjects with muscular TMD, both in terms of the global assessment and the individual evaluation by type of pain and muscle. However, care should be taken when assessing myofascial pain and the medial pterygoid muscle. This test is recommended in the clinical setting due to its safety, speed, low cost, and lack of dependence on the clinical examiner, although further studies are needed with different samples to assess individual aspects that may modify muscle pain.
